# Artificial intelligence survival models for identifying relevant risk factors for incident diabetes in Azar cohort population

**DOI:** 10.34172/hpp.025.43105

**Published:** 2025-05-06

**Authors:** Neda Gilani, Mohammadhossein Somi, Farzaneh Hamidi, Pasqualina Santaguida, Elnaz Faramarzi, Reza Arabi Belaghi

**Affiliations:** ^1^Department of Statistics and Epidemiology, Faculty of Health, Tabriz University of Medical Sciences, Tabriz, Iran; ^2^Liver and Gastrointestinal Diseases Research Center, Tabriz University of Medical Sciences, Tabriz, Iran; ^3^Student Research committee, Tabriz University of Medical Sciences, Tabriz, Iran; ^4^Department of Health Research Methods, Evidence, and Impact (HEI) Associate Member, Rehabilitation Science McMaster University, Hamilton, Canada; ^5^Unit of Applied Statistics and Mathematics, Department of Energy and Technology, Faculty of Natural Resources and Agricultural Sciences, Swedish University of Agriculture Sciences, Uppsala, Sweden

**Keywords:** Cohort study, Diabetes mellitus, Incidence, Random forest, Survival analysis

## Abstract

**Background:**

This study aimed to identify some risk factors associated with time to diabetes type II events using artificial intelligence (AI) survival models (SM) in a population cohort from East Azerbaijan, Iran.

**Methods:**

Data from Azar-Cohort spanning from 2014 to 2020 was analyzed using the random forest (RF) variable selection method along with Cox regression to identify the most relevant risk factors associated with diabetes. We then developed prediction models using RF survival analysis. Lasso-variable selection and RF variable selection were used to select the most important variables. The concordance index (C-index) was used to evaluate the concordance of the prediction models.

**Results:**

Our LASSO-Cox regression identified six factors to be significantly associated with diabetes: age, mean corpuscular hemoglobin concentration (MCHC), waist circumference (WC), body mass index (BMI), use of sleep medication, and hypertension stage 1 and stage 2. The model included all variables with a C-index of 76.3%. In contrast, the RF analysis identified 21 important variables predicting a higher probability of having diabetes. Of those, WC, MCHC, triglyceride, and age were the most important predictors of diabetes. The RF model converged after 500 trees with an out-of-bag (OOB) of 0.28 and a C-index of 79.5%.

**Conclusion:**

RF machine learning algorithms and LASSO-Cox regression analyses consistently identified WC, hypertension, and MCHC as the main risk factors for developing diabetes. The RF approach demonstrated slightly better accuracy in predicting the likelihood of diabetes at different time points.

## Introduction

 Diabetes mellitus (DM) is an umbrella term for multiple diseases with a common denominator: dysregulated blood sugar levels.^1^ Type 2 DM (T2DM) accounts for nine-tenths of all DM cases and represents the main focus of the present study.^2^ This condition typically affects adults, though the age of onset appears to be falling secondary to a complex interplay between various modifiable (e.g., lifestyle and diet) and non-modifiable (e.g., ethnicity and hereditary factors) risk factors.^3^ Roughly 372 million people were at risk of T2DM in 2019, which is projected to rise to almost half a billion by 2040.^4,5^ Following the global trend, the prevalence of T2DM in Iran saw a staggering 35% rise (7.8% to 11.9%) among adults from 2005 to 2011. By 2030, over nine million Iranians are projected to develop this condition.^6^ Furthermore, almost a third of T2DM patients in Iran lack awareness regarding their disease and its potential complications.^6^

 As T2DM leaves an immense morbidity and mortality burden,^7^ health systems constantly seek to improve their primary, secondary, and tertiary prevention methods for this disease. Therefore, it is vital to intervene now not only to deal with but also to prevent and make a well-timed detection of diabetes. Researchers have recently used machine learning algorithms to assist medical diagnostics and data mining techniques to explore risk factors.^8,9^ One example is the random forest (RF) algorithm, where multiple decision trees can be averaged to provide an accurate and robust classification of data.^8^ The algorithm produces a forest of decision trees using a random sample from the training dataset and then repeats this process with other random samples. Once an enormous forest is created, it recruits the concept of majority voting to arrive at a final decision.^9^ Hence, this study used the RF algorithm coupled with LASSO Cox proportional hazard regression (LPHR).^10^ A major strength of this approach is that it enables the researcher to select and predict variables, facilitating the most accurate diagnosis of T2DM and the classification of risk factors.

 Based on the review of previous studies, it was found that most of them determined diabetes risk factors using a categorical response model, especially the logistic regression model. Also, in some studies, the sample size was small, or the number of predictor variables included in the model was low. Another important factor is that the onset of diabetes is not considered in determining the modeling of the risk factors for diabetes. There is a pressing necessity for techniques that may effectively address these obstacles. Machine learning algorithms that can forecast the duration till a patient manifests diabetes are crucial instruments in comprehending diabetic vulnerabilities and can yield more precise outcomes compared to conventional statistical techniques. Therefore, we decided to determine the risk factors for T2DM using LASSO-Cox regression and RF algorithms. The secondary objective was to compare the performance of different predictive models in determining the relevant risk factors for T2DM. The developed models may help create suitable interventions for preventing T2DM.

## Methods

###  Study design 

 The data analyzed in this study was based on the Azar cohort study, established and collected from participants in Shabestar in East Azerbaijan province (northwest of Iran). This cohort study is also part of the state-level PERSIAN cohort (Prospective Epidemiological Research Studies in Iran) study, a prospective national cohort study of Iranian adults launched in 2014 in different geographical regions of Iran. These cohort studies aimto evaluate a wide range of biomarkers, lifestyle choices, socioeconomic status, and health-related factors associated with prevalent non-communicable illnesses in Iranian individual.^11,12^

 The Azar cohort study was launched in October 2014, and it has 4 phases (pilot, enrolment, follow-up, and re assessment).^13^ In January 2017, the enrollment phase was completed, and 15 006 subjects were recruited. The follow-up phase started in March 2017 and is progressing up to now. To follow up on individuals in the Azar Cohort study, telephone-based interviews are conducted by the research team members annually in which questions regarding death, medical events, hospitalizations, or disease diagnosis and therapy are asked. Although the study has reached its sixth follow-up, our analysis included data from three follow-ups.

###  Population cohort and diabetes definition 

 Eligible for inclusion in the Azar cohort study were individuals from 35 to 70 years of age who had resided in Shabester for at least nine months. The exclusion criteria were severe psychiatric disorders, severe physical disabilities. Exclusion criteria for the present study included subjects with diabetes, those lost to follow-up or who died, and those with missing values. Of 15 006 participants included in the enrolment phase, based on these criteria, 11 917 participants remained for analysis.

 The incidence of T2DM was determined according to data from three years of follow-up. In the follow-up phase, all participants are contacted by phone annually and asked about the occurrence of non-communicable chronic diseases. Based on the participant’s self-declaration about having diabetes, it is requested that the lab findings and prescribed medications be sent to the cohort center through virtual spaces. Finally, the disease is diagnosed based on the documentation and the opinion of two internal specialists.

###  Demographic characteristics of participants 

 Information regarding age, sex, educational level, marital status, personal habits (smoking status and alcohol consumption), sleep habits, and family history of chronic diseases were collected by well-designed questionnaires.

 We used multiple correspondence analysis (MCA) to determine the socioeconomic status of each individual according to their wealth score index (WSI). This index considers durable assets (e.g., laptops and vehicles), housing (e.g., ownership status, number of rooms), and education.

###  Measurements

 The anthropometric factors that were included in the models are weight (kg), height (cm), hip circumference (HC), waist circumference (WC), and body mass index (BMI) (calculated by dividing weight (in kilograms) by the square of height (in meters)). The blood pressure was recorded on four occasions within a single day. Each measurement was taken two minutes apart, and both arms were measured twice. The measurements were taken while the participant was sitting down, following a period of 10 minutes of rest. A skilled nurse used a mercury sphygmomanometer manufactured by Rudolf Richter, with the model number DE-72417, from Germany. The mean values of these two measurements were designated as systolic and diastolic blood pressure (SBP and DBP). To define hypertension, we followed the ACC/AHA guidelines: the use of antihypertensives, an SBP ≥ 130 mm Hg, or a DBP ≥ 80 mm Hg. We classified the blood pressure as normal, elevated, stage I, or stage II.^14^

 Blood samples were collected after a 12-hour overnight fast. Complete blood count (CBC), fasting blood sugar (FBS), creatinine, serum triglyceride (TG), high-density lipoprotein (HDL), liver enzymes (aspartate aminotransferase [AST], alanine aminotransferase [ALT], alkaline phosphatase [ALP], gamma-glutamyl transferase [GGT]) were measured by standard laboratory methods. Low-density lipoprotein (LDL) was calculated using Friedewald’s formula.^15^

###  Statistical analysis

 Descriptive statistics are summarized as frequencies and percentages for categorical variables (e.g., gender, disease, behavior, and location) and as the means (standard deviations) and median (minimum, maximum) for continuous variables. An independent t-test and chi-square test were used to compare the average and percentage of the measurements for the T2MD versus non-T2DM group. A P-value less than 0.05 was considered statistically significant.

###  Prediction models

 In the present study, Cox, Lasso-Cox, Lasso RF, and RF models were used to identify the predictors of incidence of diabetes in the Azar cohort population. The performance of these models was compared using concordance index (C-index), relative prediction error, and prediction error curve.

 The optimal model is the one that generates the highest C-index. The C-index measures rank correlation between predicted risk scores and observed time points closely related to Kendall’s τ. A C-index between 70 and 100 is a good prediction model.^16^

 Moreover, the prediction error curves (Brier score) were calculated to estimate the prediction performance at any given time. We have used the pec package in R to calculate the prediction error rate. The random Forest SRC R-package^17^ was used to develop the aforementioned procedure. Finally, to calculate the overall prediction accuracies of the RF model, we obtained the relative prediction error of the reach method compared to the RF as follows:


$$Relative\;prediction\;accuracy\;of\;a\;model=\frac{mean\;of\;the\;prediction\;error\;at\;all\;times\;of\;a\;model}{mean\;of\;the\;prediction\;error\;at\;all\;times\;of\;Random\;Forest}$$

The details of the model development for variable selection and prediction are given below:

###  Model development and assessment

 We randomly divided the data into two independent sets: 70% for training and 30% for testing the model’s performance. At first, we selected the most important variables using the LASSO-Cox regression model using the *glmnet* package in R version 4.0.5.^18^ We set 10-fold cross-validation to obtain the shrinkage parameter in the Cox model to estimate the optimal model in the training data.

 Similar to the LASSO-Cox regression model, we set the 10-fold cross variation to find the optimal RF model (with the best mtry value) in the training data. Then, we choose the positive and importance variables to substitute in the final RF model. Finally, in the testing data, we follow the guidelines for developing transparent multivariable prediction models.^19^

## Results

 We identified 104 predictor variables. After removing the missing data and refining the data, 23 variables were selected as the candidate variables for the prediction model. Accordingly, an analysis was made of data pertaining to 11 917 participants, among whom 316 (192 females and 124 males) were newly diagnosed with T2DM during the three years of follow-up.

###  General characteristics of population 

Table 1 provides a summary comparison of the variables used in the modeling. The number of females in the non-diabetic group was 6,334 (54.6%), and in the diabetic group was 192 (60.8%). The mean age in the non-diabetic group was 48.8 ± 9.14, and in the diabetic group was 52.4 ± 8.33 years; the overall sample mean age was 48.9 ± 9.14 years.

**Table 1:** General characteristics of the Azar cohort population

**Variable**	**Non-diabetes** **(n=11601)**	**Diabetes** **(n=316)**	**Overall** **(N=11917)**	***P*** value^*^
Time to event (month)				< 0.001
Mean (SD)	50.6 (7.14)	26.5 (11.7)	49.9 (8.25)	
Median [Min, Max]	51.0 [24.0, 67.0]	27.0 [1.00, 52.0]	51.0 [1.00, 67.0]	
Age (years)				< 0.001
Mean (SD)	48.8 (9.14)	52.4 (8.33)	48.9 (9.14)	
Median [Min, Max]	48.0 [35.0, 70.0]	52.0 [35.0, 70.0]	48.0 [35.0, 70.0]	
Education years (years)				< 0.001
Mean (SD)	6.52 (4.61)	5.28 (4.52)	6.48 (4.61)	
Median [Min, Max]	5.00 [0, 31.0]	5.00 [0, 18.0]	5.00 [0, 31.0]	
Mean corpuscular hemoglobin concentration (g/dL)				0.437
Mean (SD)	33.9 (0.987)	34.0 (1.18)	33.9 (0.993)	
Median [Min, Max]	33.9 [25.5, 47.6]	33.9 [29.9, 46.8]	33.9 [25.5, 47.6]	
Missing	3 (0.0%)	0 (0.0%)	3 (0.0%)	
Creatinine (mg/dL)				0.938
Mean (SD)	1.02 (0.155)	1.02 (0.165)	1.02 (0.156)	
Median [Min, Max]	1.00 [0.0830, 3.81]	0.990 [0.540, 1.81]	1.00 [0.0830, 3.81]	
Missing	3 (0.0%)	0 (0.0%)	3 (0.0%)	
Triglyceride (mg/dL)				< 0.001
Mean (SD)	144 (75.9)	178 (92.4)	145 (76.6)	
Median [Min, Max]	120 [15.0, 1150]	151 [49.0, 607]	121 [15.0, 1150]	
Missing	3 (0.0%)	0 (0.0%)	3 (0.0%)	
Cholesterol (mg/dL)				< 0.001
Mean (SD)	193 (38.9)	206 (41.3)	193 (39.0)	
Median [Min, Max]	190 [54.0, 543]	205 [112, 336]	190 [54.0, 543]	
Missing	3 (0.0%)	0 (0.0%)	3 (0.0%)	
Aspartate aminotransferase (U/L)				< 0.001
Mean (SD)	22.1 (9.47)	25.3 (14.8)	22.2 (9.66)	
Median [Min, Max]	20.0 [2.00, 239]	21.5 [11.0, 134]	20.0 [2.00, 239]	
Missing	3 (0.0%)	0 (0.0%)	3 (0.0%)	
Alanine Aminotransferase (U/L)				< 0.001
Mean (SD)	24.1 (13.8)	28.4 (17.7)	24.2 (13.9)	
Median [Min, Max]	21.0 [2.00, 442]	24.0 [10.0, 162]	21.0 [2.00, 442]	
Missing	3 (0.0%)	0 (0.0%)	3 (0.0%)	
Alkaline Phosphatase (U/l)				< 0.001
Mean (SD)	186 (55.6)	203 (64.7)	187 (55.9)	
Median [Min, Max]	179 [64.0, 912]	192 [93.0, 720]	179 [64.0, 912]	
Missing	3 (0.0%)	0 (0.0%)	3 (0.0%)	
High density lipoprotein cholesterol (mg/dL)				0.143
Mean (SD)	46.4 (11.0)	45.5 (11.3)	46.4 (11.0)	
Median [Min, Max]	45.0 [13.0, 113]	44.0 [21.0, 112]	45.0 [13.0, 113]	
Missing	3 (0.0%)	0 (0.0%)	3 (0.0%)	
Low density lipoprotein cholesterol (mg/dL)				< 0.001
Mean (SD)	117 (33.5)	125 (35.4)	118 (33.6)	
Median [Min, Max]	115 [16.0, 400]	125 [37.0, 239]	116 [16.0, 400]	
Missing	55 (0.5%)	2 (0.6%)	57 (0.5%)	
Gamma glutamyl transferase (U/l)				< 0.001
Mean (SD)	23.4 (18.2)	30.1 (25.4)	23.6 (18.5)	
Median [Min, Max]	19.0 [1.00, 530]	24.0 [7.00, 280]	19.0 [1.00, 530]	
Missing	38 (0.3%)	1 (0.3%)	39 (0.3%)	
Waist circumference (cm)				< 0.001
Mean (SD)	93.3 (11.1)	101 (11.2)	93.5 (11.2)	
Median [Min, Max]	93.2 [49.4, 153]	101 [61.5, 134]	93.5 [49.4, 153]	
Missing	6 (0.1%)	0 (0.0%)	6 (0.1%)	
Hip circumference (cm)				< 0.001
Mean (SD)	104 (8.68)	107 (9.70)	104 (8.72)	
Median [Min, Max]	104 [65.5, 158]	106 [74.3, 138]	104 [65.5, 158]	
Missing	6 (0.1%)	0 (0.0%)	6 (0.1%)	
Body mass index (kg/m^2^)				< 0.001
Mean (SD)	28.6 (4.88)	31.3 (5.01)	28.6 (4.91)	
Median [Min, Max]	28.3 [0, 56.4]	30.8 [18.4, 47.0]	28.4 [0, 56.4]	
Wealth score index				0.029
Mean (SD)	0.0274 (0.996)	-0.102 (1.03)	0.0240 (0.998)	
Median [Min, Max]	0.202 [-4.11, 3.02]	-0.231 [-4.14, 2.66]	0.202 [-4.14, 3.02]	
Sleep duration (h/d)				0.734
Mean (SD)	7.26 (1.39)	7.29 (1.38)	7.26 (1.39)	
Median [Min, Max]	7.25 [0, 13.5]	7.38 [0, 11.5]	7.25 [0, 13.5]	
Missing	2 (0.0%)	0 (0%)	2 (0.0%)	
Gender				0.034
Female	6334 (54.6%)	192 (60.8%)	6526 (54.8%)	
Male	5267 (45.4%)	124 (39.2%)	5391 (45.2%)	
Using sleeping pills				< 0.001
No	11104 (95.7%)	280 (88.6%)	11384 (95.5%)	
Yes	495 (4.3%)	36 (11.4%)	531 (4.5%)	
Missing	2 (0.0%)	0 (0.0%)	2 (0.0%)	
Smoking				0.168
No	9037 (77.9%)	257 (81.3%)	9294 (78.0%)	
Yes	2560 (22.1%)	59 (18.7%)	2619 (22.0%)	
Missing	4 (0.0%)	0 (0%)	4 (0.0%)	
Exposed to smoke in childhood				0.424
No	6285 (54.2%)	164 (51.9%)	6449 (54.1%)	
Yes	5312 (45.8%)	152 (48.1%)	5464 (45.9%)	
Missing	4 (0.0%)	0 (0%)	4 (0.0%)	
Alcohol consumption				0.549
No	10115 (87.2%)	280 (88.6%)	10395 (87.2%)	
Yes	1482 (12.8%)	36 (11.4%)	1518 (12.7%)	
Missing	4 (0.0%)	0 (0%)	4 (0.0%)	
Hypertension classification				< 0.001
Elevated	835 (7.2%)	28 (8.9%)	863 (7.2%)	
Normal	7338 (63.3%)	141 (44.6%)	7479 (62.8%)	
Stage1	2595 (22.4%)	103 (32.6%)	2698 (22.6%)	
Stage2	827 (7.1%)	44 (13.9%)	871 (7.3%)	
Missing	6 (0.1%)	0 (0%)	6 (0.1%)	

* For continuous variables based on independent sample t-test and for categorical variables based on chi-square test.

**Figure 1:**
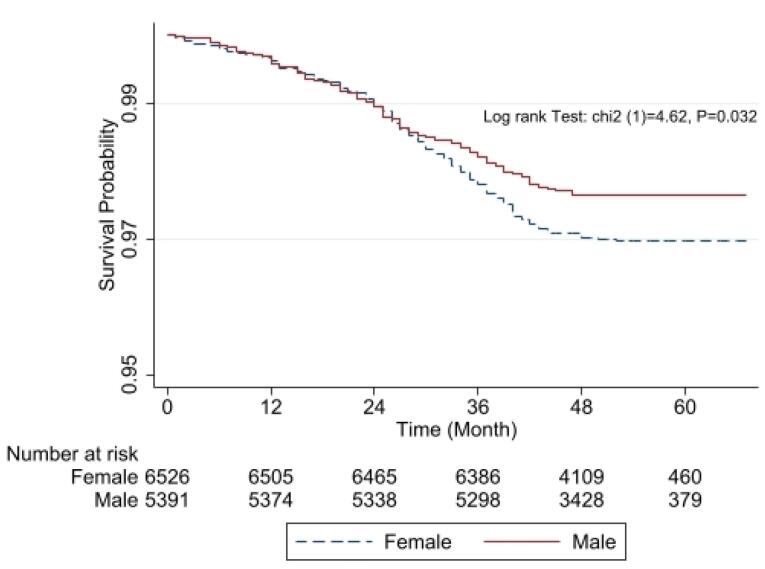
Kaplan-Meier survival curves comparing males (solid red line) and females (dashed blue line). The x-axis represents time in months, while the y-axis shows the estimated survival probability. The log-rank test indicates a statistically significant difference between the two groups (χ^2^ = 4.62, *P* = 0.032). The number at risk at different time points is displayed below the x-axis

 Those with diabetes had higher TG, CHOL, LDL, AST, ALT, ALP, and GGT levels and greater mean WC, HC, and BMI values (*P* < 0.001) than non-diabetics. The use of sleeping pills was also more prominent among the latter population (*P* < 0.001). The results of the chi-square test indicated that compared to non-diabetic subjects, diabetic subjects had significantly (*P* < 0.001) more hypertension (29.5% vs 46.5%). The Kaplan–Meier curves for the gender differences are shown in Figure 1. Figure 1 illustrates the Kaplan-Meier survival estimates stratified by gender. The survival probability decreases over time for both groups, with females experiencing a slightly higher decline compared to males. The risk table below the graph shows the number of individuals still at risk at different time points, highlighting the decreasing sample size over time. The log-rank test result (*P* = 0.032) suggests that the difference in survival between males and females is statistically significant. According to Table 1, we found that educated (*P* < 0.001) and well-off (*P* = 0.029) participants had a significantly lower incidence of diabetes than others.

###  Findings of prediction models 

 The findings of Lasso-Cox models indicated that each 1-unit increment of age, mean corpuscular hemoglobin concentration (MCHC), WC, HC, and BMI were significantly associated with a 2%, 16%, 4%, 4%, and 7% elevated risk of diabetes, respectively. We observed that using sleeping pills increased the risk of diabetes by 2.26 (95% CI: 1.58-3.22). Another finding of this study showed that hypertension increased the risk of diabetes. In this regard, the risk of diabetes in participants with hypertension stage 1 and stage 2 increased by 1.37 (95% CI: 1.05-1.79) and 1.55 (95% CI: 1.08-2.21), respectively, as compared with those without hypertension (Table 2). The LASSO-Cox regression model with all the variables had a C-index of 76.3%.

**Table 2:** Hazard ratio of variables in prediction of diabetes events

**Variable**	**Hazard ratio**	**Lower bound 95% confidence interval**	**Upper bound 95% confidence interval**	***P*** ** value**
Age	1.02	1.01	1.04	0.006
Education years	0.99	0.96	1.03	0.632
Mean corpuscular hemoglobin (gr/dL)	1.16	1.04	1.31	0.011
Creatinine (mg/dL)	0.84	0.38	1.89	0.678
Triglyceride (mg/dL)	1.00	0.99	1.01	0.988
Cholesterol (mg/dL)	1.01	0.96	1.06	0.640
Aspartate aminotransferase (U/l)	1.01	1.00	1.02	0.100
Alanine aminotransferase (U/l)	1.00	0.99	1.01	0.942
Alkaline phosphatase (U/l)	1.00	1.00	1.00	0.378
High density lipoprotein cholesterol (mg/dL)	0.99	0.94	1.04	0.583
Low density lipoprotein cholesterol (mg/dL)	0.99	0.94	1.04	0.723
Gamma-glutamyl transferase (U/l)	1.00	1.00	1.01	0.092
Waist circumference	1.04	1.02	1.06	< 0.001
Hip circumference	1.04	1.03	1.5	< 0.001
Body mass index (kg/m^2^)	1.07	1.01	1.13	0.017
Wealth score index	0.99	0.87	1.12	0.866
Sleep duration	1.02	0.94	1.09	0.679
Sex (Female)	1.39	0.97	2.00	0.073
Using sleeping pills (Yes)	2.26	1.58	3.22	< 0.001
Smoking (Yes)	1.17	0.81	1.69	0.392
Exposed to smoke in childhood (Yes)	1.14	0.91	1.43	0.255
Alcohol consumption (Yes)	1.07	0.71	1.61	0.753
Hypertension elevated	1.17	0.77	1.78	0.470
Hypertension stage 1	1.37	1.05	1.79	0.020
Hypertension stage 2	1.55	1.08	2.21	0.017

**Figure 2:**
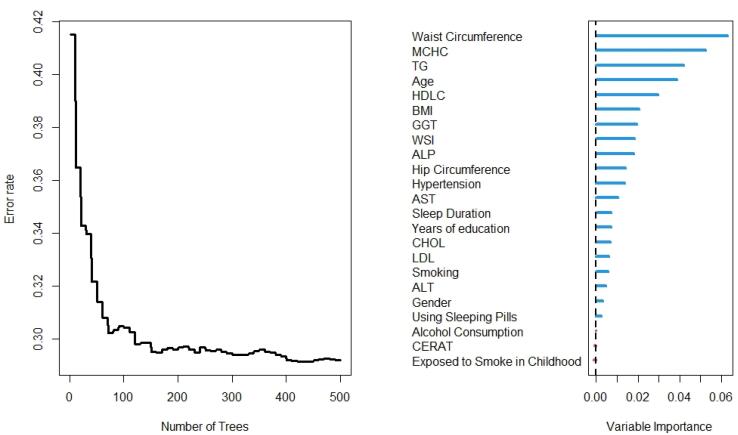
Number of trees and variables from the Random Forest for all variables

**Figure 3:**
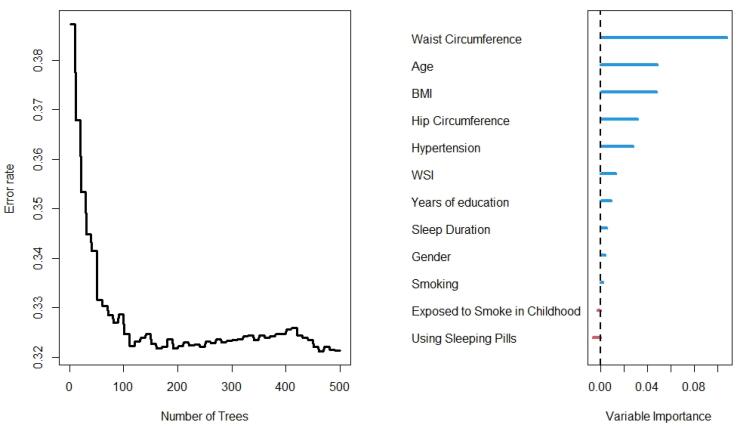
Number of trees from the Random Forest for characteristics variables

**Figure 4:**
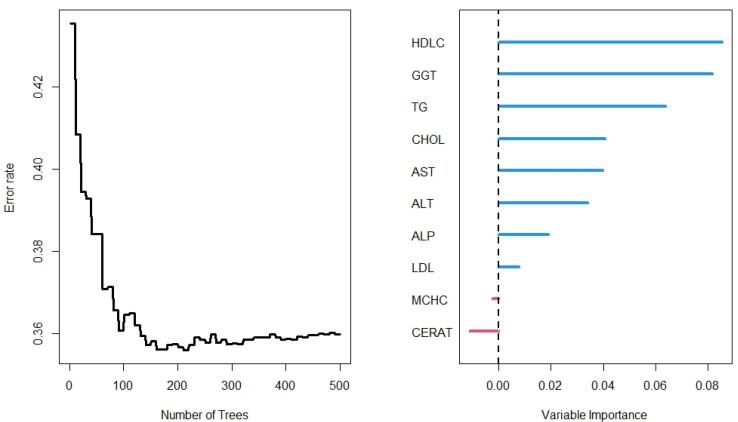
Number of trees and variable of the Random Forest for laboratory variables

 The results of the RF analysis of factors that predict the occurrence of T2DM are summarized in Figures 2-4. While Figure 2 includes all 104 variables, the models in Figures 3 and 4 included only demographic characteristics and biochemical factors, respectively.

 In this regard, RF identified 21 important variables to predict the higher probability of T2DM (Figure 2). Of these 21 variables, WC, MCHC, TG, and age were identified as the most important predictors. The random model converged in 500 trees with an out-of-bag (OOB) of 0.28 and a C-index of 79.5%. Moreover, it is observed that the WC is the most important variable, followed by age and BMI, for the prediction of diabetes (Figure 3). Using the characteristics variables, the OOB for the prediction time of diabetes was 32% after 500 iterations with a C-index of 73.2%.

 In the lab results (Figure 4), however, the RF importance results differed from other models. Unlike the full model, MCHC was not identified as an important predictor of T2DM in the presence of the laboratory variables (Figure 2). The RF models identified HDLC and GGT as the most important variables. Other important variables were TG, CHOL, AST, ALT, and ALP. The OOB was 36% for the lab results for predicting time to diabetes after 500 iterations, with a C-index of 71.2%.

**Figure 5:**
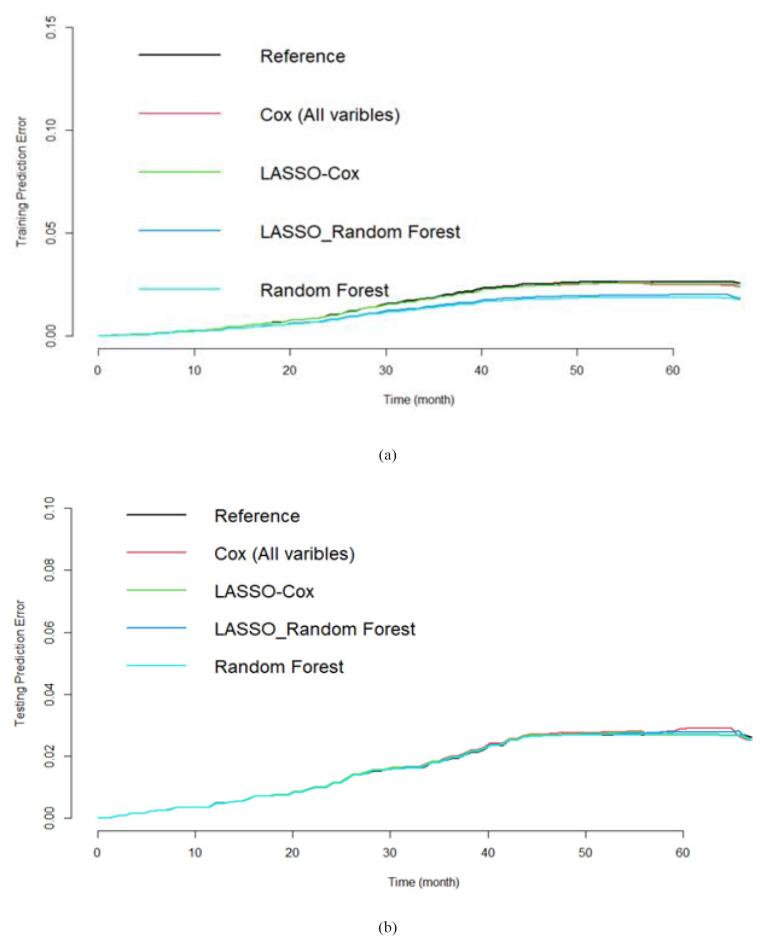
Prediction performance in the training (a) and testing (b) data sets. The reference model is the Kaplan-Meir without any covariate

 The Lasso-RF and the RF prediction errors with selected variables were lower than the reference model (Kaplan-Meier) and Cox model with all variables. However, the survival predictions of the models were close to each other (Figure 5). Finally, the survival prediction accuracy of the proposed models relative to the RF is given in Table 3. It is observed that the RF provided less mean prediction error compared to the LASSO-Cox, Cox model with all variables, Kaplan-Meier estimate, and the RF with selected variables by the LASSO-Cox. We can easily conclude that, on average, the RF provided higher accuracy than the other models. We have similar results for the laboratory variables and demographic characteristics (the data are not shown here to save space).

**Table 3:** Comparing the relative prediction error of each method compare to the random forest

**Relative mean prediction error of all time (compared to the random forest**)	**Kaplan-Meier**	**Cox model with all variables**	**LASSO-Cox**	**Random forest with Lasso selected variables**	**Random forest with its selected variables**
	1.04	1.03	1.01	1.01	1

## Discussion

 Alternative models for analyzing time-to-event outcomes, such as RF and Cox regression analyses, are becoming increasingly popular. The present study aimed to identify risk factors associated with the incidence of T2DM in the Azar cohort population. This study contributes to the existing literature by applying machine learning techniques to a large real-world dataset, considering more than one hundred variables over a three-year follow-up period in a population-based cohort study

 According to the findings from our regression and RF (full model) analysis, an increment in MCHC increased the T2DM risk; this follows the results of another study, where individuals with poor glycemic control diabetes had significantly higher MCV and MCHC levels than those with good glycemic control diabetes.^20^ However, the literature mostly reports that the RBC, MCV, and MCHC levels are lower in people with diabetes, associated with a greater prevalence of anemia.^21,22^ This discrepancy may be due to the cross-sectional nature of the mentioned reports, whereas the present analysis used data from a prospective cohort study.

 The literature also indicates that the MCHC and red blood cell distribution width (RDW) parameters are markers of inflammation, which can be triggered by elevated blood sugar and insulin resistance.^23^ Hence, the MCHC level might be able to predict the risk of T2DM development.

 Our full RF model revealed that WC, MCHC, TG, age, HDL, BMI, and GGT could strongly predict the incidence of T2DM in our study population, with WC, age, and BMI representing the strongest predictors among the demographic, anthropometric, and sleep characteristics included in model 2. Both models detected WC, age, and BMI as predictors of T2DM development. In line with our results, Xu et al cited the WC and waist-to-hip ratio as stronger predictors of T2DM than BMI,^24^ and Jeon et al noted that WC could strongly predict this disease.^25^ Another study also found that elevated TG, WC, and lipid accumulation increase the risk of T2DM.^26^

 Semerdjian et al^27^ used available samples from the NHANES 1999 to 2004 dataset. They identified the highest risk factors based on a RF analysis. Classifiers LR, KNN, RF, Gradient boosting (GB), and RF were adopted to predict the T2DM based on the 16 attributes (such as age, gender, education, BMI, weight, height, and physical activities) and the performance of GB based classifier was higher (AUC: 0.84) compared to others. Maniruzzaman et al^28^ evaluated data from the National Health and Nutrition Examination Survey conducted between 2009–2012. The dataset consisted of 657 diabetics. The LR model demonstrates that 7 factors out of 14 (age, education, BMI, systolic BP, diastolic BP, direct cholesterol, and total cholesterol) are the risk factors for T2DM. The overall ACC of the machine learning-based system is 90.62%. The combination of LR-based feature selection and RF-based classifier gives 94.25% ACC and 0.95 AUC.

 In another study, Zou et al determined predictive risk factors for diabetes using machine learning techniques in two different data sets.^29^ They found that FBS, weight, and age were the important risk factors for diabetes in the Luzhou dataset. Also, blood sugar, age, and insulin play an important role in the Pima Indians dataset. Similarly, findings of another study indicated that based on different statistical methods, blood glucose, and BMI are strongly associated with diabetes.^30^

 An increased WC is linked with greater intra-abdominal fat, with free fatty acids being released more into the systemic circulation and inducing hyperinsulinemia and insulin resistance.^31^

 Furthermore, we found a link between liver enzymes and diabetes, with GGT being a remarkably stronger T2DM predictor than AST, ALT, and ALP. Other reports from the literature have also indicated that liver enzymes positively correlate with the occurrence of diabetes. Moreover, the greater predictive value of GGT has also been seen in prior investigations, which noted this enzyme as an independent risk factor for T2DM development.^32-34^

 The exact mechanism to explain the relationship between liver enzymes and T2DM risk remains elusive. However, it is presumed that such liver enzyme elevations may indicate the occurrence of NAFLD,^35^ which is strongly linked with T2DM.^36^

 In line with our second objective, which was to compare the performance of different predictive models, we confirmed that the RF model was better than the other models. Comparison of the proposed work with similar research works and state-of-the-art research studies indicated that the performance of predictive models in various studies was assessed by different indices. In this regard, Semerdjian et al^27^ reported that the Gradient Boosting Classifier performs best with an AUC of 0.84. In another study, it has been reported that the combination of LR-based feature selection and RF-based classifier gives 94.25% ACC and 0.95 AUC.

 The major differences between our results and aforementioned studies are the low number of predictors and the lack of consideration of the nature of the time until the onset of diabetes.

## Strengths and limitations of the study

 One of the key advantages of using machine learning models, such as RF, is their ability to capture non-linear relationships and complex patterns in the data. Our study leveraged a large population-based cohort with more than 100 variables, allowing for a comprehensive analysis of potential risk factors. Additionally, the prospective design of our study provides stronger evidence compared to many previous studies that were cross-sectional.

 One limitation of the current study is the high proportion of censored observations. Even with the employment of the advanced RF model, the C-index is at most 79%, which indicates that the prediction accuracy can be improved. We suggest that future studies implement deep learning approaches. Additionally, a limitation of this study is that the diagnosis of T2DM was determined by self-report rather than confirmation using health records.

 The generalizability of our findings may be influenced by several factors. First, our study is based on data from the Azar cohort, which represents a specific population with its own demographic and lifestyle characteristics. While the use of a machine learning approach, such as RF, enhances the model’s adaptability, external validation in different populations is necessary to confirm its broader applicability. Additionally, variations in healthcare systems, genetic backgrounds, and environmental exposures could impact the model’s performance when applied to other populations. Future studies should focus on testing our approach in diverse cohorts to assess its robustness and reliability across different settings.

## Conclusion

 In this study, we implemented advanced RF machine-learning approaches for variable selection and prediction. We compared the results with the traditional statistical approaches. The results showed that RF models had slightly better accuracy than the traditional approaches. Considering the high accuracy of forest models compared to other models, the findings of this model indicated that WC, MCHC, TG, HDL, BMI, GGT, age, and BMI are the best predictors of the onset of diabetes. In other models of a RF model, when only demographic or lab findings entered the model, the strong factors were WC, age, BMI, WC, HDL-C, GGT, TG, CHOL, AST, ALT, and ALP. Because measurements of these parameters are relatively simple to perform in a clinical setting, considering these factors to identify individuals at high risk of T2DM has important public health implications for early prevention and treatment.

## Competing Interests

 The authors declare that the research was conducted without any commercial or financial relationships that could be construed as a potential conflict of interest.

## Data Availability Statement

 The data are available in the Azar Cohort website: https://irancohorts.ir/azar-cohort-study/

## Ethical Approval

 This study was approved by the Ethics Committee of Tabriz University of Medical Sciences (IR.TBZMED.REC.1400.429). Written informed consent was obtained from all participants.
